# Salicylate of Mercury in Venereal and Cutaneous Diseases

**Published:** 1888-06

**Authors:** 


### SALICYLATE OF MERCURY IN VENEREAL AND CUTANEOUS DISEASES.

Lajoux and Grandval, of Rheims, were the first to make a chemical study of salicylate of mercury, in 1881, their observations being published in the following year, in the *Journal de Pharmacie et de Chemie.* In 1886 a Brazilian student, A. Duprat, presented to the Société de Biologie de Paris a note on the physiological action of salicylate of mercury, in which he reported some experiments made with this salt on frogs. In the same year Professor Silva Araujo, of Rio de Janeiro, made a clinical study of the salt, and within a little more than a year about a dozen communications from his pen have appeared on the same subject.

In April, 1887, Dr. Bruno-Chaves, of Rio de Janeiro, wrote in the *Medical and Surgical Reporter* of Philadelphia, in regard to the use of salicylate of mercury in syphilides: "Our success exceeded our expectations, and from what I have observed I am convinced that the salicylate of mercury will become as esssential to therapeutics as the iodide of potassium, calomel, and other useful medicines." From the more recent reports on the subject it appears that he did not over-rate the value of the drug. Its value in ophthalmology has been tested and confirmed by such men as Moura Brazil and Neves da Rocha, Lara, and Guedes de Mello. Ribeiro dos Santos, of Bahia, whose clinic is very large, speaks highly of the drug in conjunctivitis and iritis of syphilitic origin.

Of its physiological action Duprat says: We may conclude that it acts on the central bulbo-medullary system, the action seeming to be that of the components, mercury and salicylic acid. Joâo Paulo, of Rio de Janeiro, confirms Araujo's observations and results with the drug, and says that it is a more powerful drug than the phenate of mercury. He has had excellent results in administering it to syphilitics in increasing doses, from 5 millig. to 5 centig., preferably in the form of pills.

Bruno-Chaves recommends the following formula, known as Delgado's unalterable mercurial pills:

| | |
|---|---|
| Salicylate of mercury.... | 0.01 centig. |
| Extract of carobe........ | 0.05 centig. |
| Extract of licorice............. | q. s. |
| Licorice powder................ | q. s. |

This is the formula for one pill; 2, 3, or 4 of these are to be taken a day in the more common cases. They may be covered with some protective substance, or simply silvered.

Mixed with vaseline the salicylate of mercury may be used externally. It unites intimately with fatty bodies, and forms a very homogeneous mass with them. Administered topically, in doses of 50 centig. up to 2 grams of the salicylate to 30 grams of white vaseline, the drug has an excellent effect in some syphilitic affections. The drug has been used also for subcutaneous injections by Moura Brazil and Neves da Rocha, in the proportion of from 5 to 10 millig. to 10 grams of distilled water.

For injections into the urethra the following solution has given good results:

| | |
|---|---|
| Salicylate of mercury..... | 0.15 centig. |
| Rose water ............. | 250 grams. |
| Carbonate of soda ............. | q. s. |

To be given in three injections a day.

This formula may be used also as a collyrium, as has been done by Moura Brazil and Ribeiro dos Santos.

In a large number of cases in which the drug was used internally by Bruno-Chaves in one case only was even slight intestinal irritation observed, nor does it produce stomatitis, unless taken in very large doses.

But the good effects of the drug are not confined to syphilitic cases. Silva Araujo says that in the parasitic dermatoses (eczema marginata, pityriasis circinata, parasitic sycosis, pityriasis versicolor, tinea favosa, tinea tonsurans) it has an energetic action second to none of the parasiticides in general use; and it has the advantage of being odorless and unirritating when the doses are proportioned to the intensity of the disease to be combatted. One Brazilian dermatologist reports a cure of a case of trichorrexis nodosa (trichoptilosis) affecting the moustache.

Bruno-Chaves reports good results from its use in lepra, used in connection with gynocardic acid (the active principle of oil of gynocardia, or chaulmoogra oil). The patients recovered normal sensibility, and the leprous patches disappeared. It is true that the cases were mild, but there has been no recurrence. Silva Araujo speaks highly of its action in lepra. Bruno-Chaves has used the gynocardate of mercury in leprosy, in pills of 0.05 grams, made with .10 centig. of extract of gentian, and a sufficient quantity of powdered licorice.

Moura Brazil was the first to use the drug in diseases of the eye.

He gives it internally in the form of pills, and in his very large practice he has not found a single patient whose stomach refused to tolerate them. He has had the best possible result in 45 cases of iritis, 50 cases of choroiditis, 10 of scleritis, 25 of retinitis, 8 of muscular paralysis, and 3 of neuro-retinitis. He has had good results in a case of papillary atrophy of syphilitic origin, in lymphatic keratitis, in pustular conjunctivitis, and in impetiginous eczema of the pupil. In purulent conjunctivitis, the drug has a great advantage over nitrate of silver, since it causes less pain, and does not stain the hand of the physician. In simple conjunctivitis Moura Brazil uses a solution of 50 centig. to 30 grams of distilled water; in blennorraghic conjunctivitis the solution is 1 gram of the salt to 30 grams of water.—*Annales de Dermatologie et de Syphilographie, April* 25, 1888.

